# Use of natural language processing to improve predictive models for imaging utilization in children presenting to the emergency department

**DOI:** 10.1186/s12911-019-1006-6

**Published:** 2019-12-30

**Authors:** Xingyu Zhang, M. Fernanda Bellolio, Pau Medrano-Gracia, Konrad Werys, Sheng Yang, Prashant Mahajan

**Affiliations:** 1 Department of Systems, Populations and Leadership, University of Michigan School of Nursing, Ann Arbor, USA; 2 Department of Emergency Medicine, Mayo Clinic, Rochester, USA; 3 Department of Anatomy and Medical Imaging, University of Auckland, Auckland, New Zealand; 4 Oxford Centre for Clinical Magnetic Resonance Research, University of Oxford, Oxford, UK; 5 Department of Biostatistics, School of Public Health, Nanjing Medical University, Nanjing, China; 6 Department of Biostatistics, University of Michigan School of Public Health, Ann Arbor, USA; 7 Department of Emergency Medicine, University of Michigan School of Medicine, Ann Arbor, USA

**Keywords:** Pediatric emergency department, Natural language processing, Predictive model, Medical imaging utilization

## Abstract

**Objective:**

To examine the association between the medical imaging utilization and information related to patients’ socioeconomic, demographic and clinical factors during the patients’ ED visits; and to develop predictive models using these associated factors including natural language elements to predict the medical imaging utilization at pediatric ED.

**Methods:**

Pediatric patients’ data from the 2012–2016 United States National Hospital Ambulatory Medical Care Survey was included to build the models to predict the use of imaging in children presenting to the ED. Multivariable logistic regression models were built with structured variables such as temperature, heart rate, age, and unstructured variables such as reason for visit, free text nursing notes and combined data available at triage. NLP techniques were used to extract information from the unstructured data.

**Results:**

Of the 27,665 pediatric ED visits included in the study, 8394 (30.3%) received medical imaging in the ED, including 6922 (25.0%) who had an X-ray and 1367 (4.9%) who had a computed tomography (CT) scan. In the predictive model including only structured variables, the *c*-statistic was 0.71 (95% CI: 0.70–0.71) for any imaging use, 0.69 (95% CI: 0.68–0.70) for X-ray, and 0.77 (95% CI: 0.76–0.78) for CT. Models including only unstructured information had *c*-statistics of 0.81 (95% CI: 0.81–0.82) for any imaging use, 0.82 (95% CI: 0.82–0.83) for X-ray, and 0.85 (95% CI: 0.83–0.86) for CT scans. When both structured variables and free text variables were included, the *c*-statistics reached 0.82 (95% CI: 0.82–0.83) for any imaging use, 0.83 (95% CI: 0.83–0.84) for X-ray, and 0.87 (95% CI: 0.86–0.88) for CT.

**Conclusions:**

Both CT and X-rays are commonly used in the pediatric ED with one third of the visits receiving at least one. Patients’ socioeconomic, demographic and clinical factors presented at ED triage period were associated with the medical imaging utilization. Predictive models combining structured and unstructured variables available at triage performed better than models using structured or unstructured variables alone, suggesting the potential for use of NLP in determining resource utilization.

## Introduction

More than 25 million pediatric patients seek medical care in the Emergency Department (ED) each year in the United States, and the pediatric ED utilization continues to increase [1]. Emergency providers usually need to make quick and complex clinical decisions with limited information [2]. Clinical decision making in pediatric patients is complicated and time-consuming because of their unique physiologic and developmental differences [3, 4]. Consequently, ED health outcomes of children differ as their pattern of illness and presenting symptoms vary with age [5, 6].

In many instances clinical decision making in the ED involves ordering of laboratory tests (blood, urine tests etc.) and/or performance of imaging procedures (x-rays, ultrasound, computed tomography (CT) scans) in order to arrive at working diagnosis to initiate therapies or other interventions which in some instances are lifesaving [7]. However, use of imaging has a significant impact on emergency care delivery both in terms of appropriateness as well as the impact of such studies on patient throughput, which in turn impacts access to emergency care and overcrowding [8, 9].

Previous studies have focused on improving the efficiency and accuracy of pediatric medical decisions during ED visits [10, 11]. Utilization of predictive analytical techniques to more rapidly determine patient health outcomes among adult ED patients have proved useful [12]. However, few studies have focused on predicting resource utilization (e.g., medical imaging use) of pediatric ED patients [13]. In addition, unstructured data such as patient chief complaints often available at the time of patient visiting, and contains valuable information that can potentially enhance the prediction performance [14, 15]. However, these data are not immediately useful and require extraction, cleaning, and aggregation [16]. Our previous work has revealed that the incorporation of unstructured clinical notes can increase predictive accuracy for adult hospital admission using natural language processing [12] .

Early and accurate prediction of the need for medical imaging in pediatric patients visiting the ED may assist in the planning and optimization of resources in the ED healthcare service. In the current study, we examined the association between the medical imaging utilization and information related to patients’ socioeconomic, demographic and clinical factors during the pediatric patients’ ED visits; and developed predictive models using these associated factors including natural language elements to predict the medical imaging utilization at pediatric ED.

## Data and methods

We used standardized guidelines for the conduction and reporting of this study including the Guidelines for Developing and Reporting Machine Learning Predictive Models in Biomedical Research [17]. We performed a secondary data analysis on the 2012–2016 National Hospital Ambulatory Medical Care Survey ED Subfile (NHAMCS-ED) [18–20]. NHAMCS is a multistage, stratified probability sample of ED visits from 300 hospital-based EDs each year, which was randomly selected from about 1900 geographically defined areas across the United States, administered by the National Center for Health Statistics. Details of the survey methodology are available from the National Center for Health Statistics [19, 20]. We included a total of 27,665 pediatric patients (≤18 years old) visits for analysis in the survey datasets from 2012 to 2016. This represents 161,340,000 ED visits on the national level including the patient visit weight.

The primary outcome variables for this study were performance of any diagnostic imaging (X-ray and/or CT), any X-Ray use, and CT scan during an ED visit. Ultrasound use was not included in the study as the frequency of ultrasound use was low in the NHAMCS-ED. Structured covariates included information routinely collected at the time of ED triage: sex, age category, race/ethnicity, type of residence, source of payment, arrival mode, arrival day and time, initial vital signs (body temperature, heart rate, respiratory rate, blood pressure, pulse oximetry), 5 point triage level (1 Immediate; 2 Emergent; 3 Urgent; 4 Semi Urgent; 5 Nonurgent), pain scale, 72 h revisit, comorbidities (cancer, cerebrovascular disease, chronic obstructive pulmonary disease (COPD), congestive heart failure, and HIV), whether the visit was related to an injury, poisoning, or adverse effect of medical treatment. Description analysis of these structured variables were performed among each medical imaging group, and the odds ratios of using any imaging, X-Ray and CT scan were estimated using logistic regression.

Unstructured data included up to three reasons for visiting the ED, and three causes of injury recorded by the providers for each patient in the triage notes [21]. Natural Language Processing (NLP) techniques were used to extract the information from the unstructured data. Firstly, we conducted a text preprocessing step which included lemmatization (grouping word capitalization and derivations together), removal of numbers, punctuations, and stop words (e.g., ‘and’, ‘are’, ‘the’), and tokenization (breaking the text into single words and word pairs). We extracted all the unigrams (single words) and bigrams (word pairs) from the free text data after preprocessing. Subsequently, the frequency of each tokenized word or word-pairs for each person or visit can be formed [22]. We finally removed sparse terms, i.e., those with a frequency lower than 99.9% of the overall population. The words or word-pairs with frequency less than 277 (1% of the total sample size 27,665) were removed. A total of 1209 words and word-pairs were identified after preprocessing the unstructured data.

For both structured data and the word (or word pairs) frequencies, principal component analysis (PCA) was used to decrease the dimension (or select the features) of the structured data and frequency table of tokenized words or word pairs. As is described in previous studies [12, 23], the goal of PCA is to obtain a fewer number of new variables, or principal components from the word or word pairs to represent large number of words or word pairs, using a linear combination. These principal components account for the maximum original variance or information of those words or word pairs. The first components derive as much of the variance in the word or word pairs frequency as possible, with each succeeding principal components accounting for the largest possible remain variance. There principal components have no information overlap between each other, based on the linear orthogonal algorithm.

Logistic regression models were used to predict the pediatric medical imaging utilization. We established three models to determine the predictive performance in identifying patients with any medical imaging use, X-Ray, or CT scan: (1) models with structured variables only; (2) models with unstructured data; (3) models with both structured and unstructured variables. Missing values were imputed with median of each corresponding variable. Ten-fold cross-validation was used to validate the performance of each model. Patients were randomly divided into 10 sets, and 9 of the 10 sets were used to train the models while the one left was used as the testing set. For each round of training, t-tests compared principal components’ scores between outcome groups. Principal components with *p* < 0.05 were used to establish the logistic regression models’ input variables.

The area under the receiver operating curve (AUC), or *c*-statistic, was recorded for each testing set. The c-statistic informs in a single numerical value about the overall diagnostic accuracy of the index test. The c-statistic ranges from 0.50 to 1.00, with higher values indicating better predictive models. Values above 0.80 indicate very good models, between 0.70 and 0.80 good models, and between 0.50 and 0.70 weak models. The average ROC curve was derived by comparing the prediction values from all 10 cross-validation testing sets. The AUCs from different models were compared using t-test. The probabilities of medical imaging use for each patient were calculated with this model. The best cutoff of the probabilities was determined by using the point on the ROC curve with the shortest distance to the upper left corner (where sensitivity = 1 and specificity = 1). The best cutoff of the probabilities for prediction and the corresponding sensitivity, specificity, and overall accuracy were recorded [24].

We performed a sensitivity analysis to predict the two major subtypes of the CT scan (abdomen/pelvis and head CT) using the same modelling strategies described above. The best cut-off of the probabilities, sensitivity, specificity, overall accuracy, and AUC were recorded. Basic data organization was done in SAS 9.4. The text analyses were performed in R 3.3.2. The modeling of logistic regression was performed in MATLAB R2016b.

## Results

Among the 27,665 ED patient visits from 2012 to 2016, 30.3% (8394/27,665) received a medical imaging, including 25.0% (*n* = 6922) who had an X-ray and 4.9% (*n* = 1367/27,665) who had a CT scan (Table 1). Male patients (31.6%) present higher imaging use than females (29.1%). Younger kids had lower proportion of receiving imaging than older (3.3% by < 1 year and 21.7% by 1–6 years, compared to 32.3% by 6–12 years and 41.4% by older than 12 years). Hispanic patients had lower imaging use (27.6%) than non-Hispanics (31.2%), and black patients presented lower imaging (26.8%) than white (32.9%) and Asian (29.4%). Patients with private insurance (36.4%) and Medicare (33.6%) had higher imaging use than patients with Medicaid (27.4%) and no insurance (29.3%). Patients with immediate or emergent triage (37.1%) and patients urgent or semi-urgent (39.2%) had higher use of imaging than non-urgent patients (25.9%). A number of 45.5% of patients with injury/trauma received medical imaging, which is higher than patients with overdose/poisoning (13.9%), patients with adverse effect of medical treatment (20.3%), and patients with no injury/trauma, overdose/poisoning, or adverse effect of medical treatment (23.4%).

**Table 1:** Baseline characteristics of U.S. patients presenting to the ED, stratified by medical imaging utilization, NHAMCS 2012–2016

	All N(%)	No Imaging N(%)	Any imaging N(%)	X-Ray N(%)	CT Scan N(%)	*P* value
	27,665	19,271(69.7)	8394(30.3)	6922(25.0)	1367(4.9)	
Sex						
Female	13,542(48.9)	9606(70.9)	3936(29.1)	3170(23.4)	625(4.6)	< 0.0001
Male	14,123(51.1)	9665(68.4)	4458(31.6)	3752(26.6)	742(5.3)	
Age category						
< 1 year	3191(12.2)	2448(76.7)	743(23.3)	653(20.5)	68(2.1)	< 0.0001
1–6 year	9447(36.2)	7400(78.3)	2047(21.7)	1829(19.4)	187(2.0)	
6–12 year	6390(24.5)	4325(67.7)	2065(32.3)	1759(27.5)	291(4.6)	
12–18 year	7033(27.0)	4118(58.6)	2915(41.4)	2271(32.3)	628(8.9)	
Ethnic						
Hispanic	6776(24.5)	4908(72.4)	1868(27.6)	1516(22.4)	272(4.0)	< 0.0001
Non-Hispanic	20,889(75.5)	14,363(68.8)	6526(31.2)	5406(25.9)	1095(5.2)	
Race						
White	14,692(68.9)	9859(67.1)	4833(32.9)	3928(26.7)	902(6.1)	< 0.0001
Black	5773(27.1)	4227(73.2)	1546(26.8)	1353(23.4)	180(3.1)	
Asian	557(2.6)	393(70.6)	164(29.4)	136(24.4)	19(3.4)	
Other	315(1.5)	231(73.3)	84(26.7)	71(22.5)	11(3.5)	
Residence						
Private residence	26,783(99.2)	18,599(69.4)	8184(30.6)	6747(25.2)	1323(4.9)	0.1114
Nursing home	36(0.1)	22(61.1)	14(38.9)	10(27.8)	7(19.4)	
Homeless	27(0.1)	20(74.1)	7(25.9)	6(22.2)	2(7.4)	
Other	156(0.6)	96(61.5)	60(38.5)	51(32.7)	12(7.7)	
Insurance						
Private insurance	7572(29.7)	4819(63.6)	2753(36.4)	2193(29.0)	543(7.2)	< 0.0001
Medicare	235(0.9)	156(66.4)	79(33.6)	61(26.0)	19(8.1)	
Medicaid or CHIP	15,448(60.6)	11,212(72.6)	4236(27.4)	3562(23.1)	569(3.7)	
Uninsured	1546(6.1)	1093(70.7)	453(29.3)	365(23.6)	81(5.2)	
Other	692(2.7)	459(66.3)	233(33.7)	200(28.9)	46(6.6)	
Arrival by Ambulance						
No	25,059(93.6)	17,562(70.1)	7497(29.9)	6219(24.8)	1093(4.4)	< 0.0001
Yes	1711(6.4)	1032(60.3)	679(39.7)	518(30.3)	247(14.4)	
Visit year						
2012	6641(24.0)	4526(68.2)	2115(31.8)	1714(25.8)	393(5.9)	< 0.0001
2013	5709(20.6)	4088(71.6)	1621(28.4)	1357(23.8)	240(4.2)	
2014	5906(21.3)	4193(71.0)	1713(29.0)	1425(24.1)	272(4.6)	
2015	4954(17.9)	3400(68.6)	1554(31.4)	1259(25.4)	262(5.3)	
2016	4455(16.1)	3064(68.8)	1391(31.2)	1167(26.2)	200(4.5)	
Visit month						
Dec-Feb	6718(24.3)	4705(70.0)	2013(30.0)	1697(25.3)	305(4.5)	0.0006
Mar-May	7055(25.5)	4866(69.0)	2189(31.0)	1820(25.8)	360(5.1)	
Jun-Aug	6745(24.4)	4815(71.4)	1930(28.6)	1562(23.2)	312(4.6)	
Sep-Nov	7147(25.8)	4885(68.4)	2262(31.6)	1843(25.8)	390(5.5)	
Day of Week						
Sunday	4109(14.9)	2902(70.6)	1207(29.4)	1018(24.8)	168(4.1)	0.3082
Monday	4478(16.2)	3117(69.6)	1361(30.4)	1110(24.8)	227(5.1)	
Tuesday	4008(14.5)	2781(69.4)	1227(30.6)	1010(25.2)	201(5.0)	
Wednesday	3805(13.8)	2668(70.1)	1137(29.9)	934(24.5)	192(5.0)	
Thursday	3808(13.8)	2613(68.6)	1195(31.4)	974(25.6)	201(5.3)	
Friday	3617(13.1)	2484(68.7)	1133(31.3)	940(26.0)	190(5.3)	
Saturday	3840(13.9)	2706(70.5)	1134(29.5)	936(24.4)	188(4.9)	
Arrival time						
Morning	5529(20.4)	3857(69.8)	1672(30.2)	1368(24.7)	272(4.9)	0.0001
Afternoon	7383(27.2)	5052(68.4)	2331(31.6)	1904(25.8)	385(5.2)	
Evening	5872(21.6)	3993(68.0)	1879(32.0)	1572(26.8)	292(5.0)	
Night	8344(30.8)	5936(71.1)	2408(28.9)	1996(23.9)	395(4.7)	
Triage level						
Immediate and Emergent	1465(7.4)	921(62.9)	544(37.1)	414(28.3)	148(10.1)	< 0.0001
Urgent and Semi-urgent	6746(33.9)	4100(60.8)	2646(39.2)	2000(29.6)	566(8.4)	
Nonurgent	11,698(58.8)	8669(74.1)	3029(25.9)	2737(23.4)	273(2.3)	
Temperature						
36 C-38 C	22,667(86.9)	15,719(69.3)	6948(30.7)	5651(24.9)	1199(5.3)	0.8118
< =36 C	684(2.6)	475(69.4)	209(30.6)	161(23.5)	39(5.7)	
> 38 C	2742(10.5)	1918(69.9)	824(30.1)	774(28.2)	43(1.6)	
Diastolic BP						
60–80	11,502(41.6)	7482(65.0)	4020(35.0)	3225(28.0)	734(6.4)	< 0.0001
< 60	13,541(48.9)	10,228(75.5)	3313(24.5)	2875(21.2)	396(2.9)	
> 80	2622(9.5)	1561(59.5)	1061(40.5)	822(31.4)	237(9.0)	
Systolic BP						
80–120	11,152(40.3)	7604(68.2)	3548(31.8)	2873(25.8)	596(5.3)	< 0.0001
< 80	10,238(37.0)	7966(77.8)	2272(22.2)	2047(20.0)	200(2.0)	
> 120	6275(22.7)	3701(59.0)	2574(41.0)	2002(31.9)	571(9.1)	
Heart Rate						
< 60	7589(27.4)	4618(60.9)	2971(39.1)	2302(30.3)	607(8.0)	< 0.0001
60–90	2687(9.7)	1979(73.7)	708(26.3)	584(21.7)	131(4.9)	
> 90	17,389(62.9)	12,674(72.9)	4715(27.1)	4036(23.2)	629(3.6)	
Pulse Oximetry						
< 95	788(3.3)	444(56.3)	344(43.7)	316(40.1)	39(4.9)	< 0.0001
> =95	23,430(96.7)	16,214(69.2)	7216(30.8)	5934(25.3)	1214(5.2)	
Pain level						
Mild	9097(54.0)	7123(78.3)	1974(21.7)	1649(18.1)	300(3.3)	< 0.0001
Moderate	4677(27.8)	2613(55.9)	2064(44.1)	1650(35.3)	364(7.8)	
Very severe	3062(18.2)	1608(52.5)	1454(47.5)	1152(37.6)	286(9.3)	
Injury/poisoning						
Injury/trauma	8963(33.5)	4886(54.5)	4077(45.5)	3551(39.6)	703(7.8)	< 0.0001
Overdose/poisoning	280(1.0)	241(86.1)	39(13.9)	37(13.2)	5(1.8)	
Adverse effect of medical/surgical treatment	296(1.1)	236(79.7)	60(20.3)	47(15.9)	17(5.7)	
Not related to any above	17,112(64.0)	13,111(76.6)	4001(23.4)	3119(18.2)	603(3.5)	
Questionable injury status	81(0.3)	66(81.5)	15(18.5)	10(12.3)	5(6.2)	
72 h Revisit						
Yes	1029(4.1)	745(72.4)	284(27.6)	223(21.7)	53(5.2)	0.0429
No	24,092(95.9)	16,728(69.4)	7364(30.6)	6053(25.1)	1201(5.0)	
Cancer						
Yes	74(0.3)	45(60.8)	29(39.2)	23(31.1)	8(10.8)	0.0974
No	27,591(99.7)	19,226	8365 (30.3)	6899 (35.9)	1359 (4.9)	
Cerebral Cardiovascular disease						
Yes	16(0.1)	6(37.5)	10(62.5)	8(50.0)	6(37.5)	0.0051
No	27,649(99.9)	19,265 (69.8)	8384(30.3)	6914(35.9)	1361 (4.9)	
Congestive Heart Failure						
Yes	8(0.1)	3(37.5)	5(62.5)	4(50.0)	1(12.5)	0.0478
No	27,657(99.9)	19,268(69.8)	8389(30.3)	6918(35.9)	1366 (4.9)	
COPD						
Yes	135(0.5)	63(46.7)	72(53.3)	70(51.9)	1(0.7)	< 0.0001
No	27,530(99.5)	19,208(69.8)	8322(30.3)	6852(35.7)	1366 (5.0)	
HIV						
Yes	17(0.1)	13(76.5)	4(23.5)	3(17.6)	1(5.9)	0.5411
No	27,648(99.9)	19,258(69.8)	8390(30.3)	6919(35.9)	1366 (5.0)	

Note: Missing value for systolic and diastolic blood pressure, arrival by ambulance, patient’s residence type, arrival time, and whether the visit is related to injury/poisoning is lower than 5%. Missing values for body temperature, heart rate, pulse oximetry, source of payment, episode of care, 72 h revisit are between 10 and 15%. Missing values for pain level is 38%, and triage level pain scale is 64%

The crude and adjusted odds ratio of ED visits resulting in different types of medical imaging (vs. no medical imaging) for each variable using binary logistic regression are presented in Table 2. Adjusted analyses showed patients between 1 and 6 years and between 6 and 12 were 42 and 24% less likely to require any medical imaging than patients less than 1 year old, respectively (aOR: 0.58, 95% CI 0.47–0.72 and aOR: 0.76, 95% CI 0.60–0.96). Black patients were 12% less likely for any imaging use than white patients (aOR: 0.88, 95% CI 0.77–1.00). Compared to those with private insurance, patients with Medicaid were 18% less likely for any imaging use than patients with private insurance (aOR: 0.82, 95% CI 0.73–0.92). Compared to those with mild pain level, patients with moderate and very severe levels were 2.15 and 2.70 more likely to receive any imaging respectively (aOR: 2.15, 95% CI 1.90–2.44 and aOR: 2.70, 95% CI 2.32–3.13). Compared to those with injury/trauma, patients with overdose/poisoning were 82% less likely to receive any imaging (aOR: 0.18, 95% CI 0.09–0.34) Patients with adverse effects of medical treatment and patients with other diagnoses were 80 and 68% less likely for any imaging use than patient with injury, respectively (aOR: 0.20, 95% CI 0.11–0.37 and aOR: 0.32, 95% CI 0.28–0.36). The odds ratios of those characteristics for X-Ray use are similar to the risk for any imaging use, as X-Ray is the most frequent medical imaging type.

**Table 2:** Adjusted odds ratio of characteristics associated with the use of diagnostic imaging studies during the emergency department visit (vs. no imaging use), NHAMCS 2012–2016

	Any imaging		X-Ray		CT Scan	
	Crude	Adjusted	Crude	Adjusted	Crude	Adjusted
Male vs Female	1.13(1.07–1.19)	1.03(0.92–1.15)	1.18(1.12–1.25)	1.01(0.91–1.13)	1.15(1.03–1.28)	1.20(0.97–1.50)
Age category						
< 1 year	Reference [1]	Reference [1]	Reference [1]	Reference [1]	Reference [1]	Reference [1]
1–6 year	0.91(0.83–1.00)	0.58(0.47–0.72)	0.93(0.84–1.03)	0.62(0.50–0.77)	0.93(0.70–1.23)	0.83(0.44–1.54)
6–12 year	1.57(1.43–1.73)	0.76(0.60–0.96)	1.48(1.33–1.64)	0.78(0.61–0.99)	2.19(1.68–2.86)	1.44(0.77–2.69)
12–18 year	2.33(2.12–2.56)	0.84(0.66–1.08)	1.85(1.68–2.05)	0.79(0.61–1.03)	4.50(3.49–5.80)	1.91(1.01–3.62)
Non-Hispanic vs Hispanic	1.19(1.12–1.27)	1.21(1.05–1.40)	1.21(1.14–1.29)	1.16(1.00–1.35)	1.32(1.16–1.52)	1.32(0.98–1.79)
Race						
White	Reference [1]	Reference [1]	Reference [1]	Reference [1]	Reference [1]	Reference [1]
Black	0.75(0.70–0.80)	0.88(0.77–1.00)	0.84(0.78–0.90)	1.03(0.90–1.18)	0.49(0.42–0.58)	0.44(0.32–0.61)
Asian	0.85(0.71–1.03)	1.29(0.94–1.76)	0.89(0.73–1.08)	1.22(0.88–1.68)	0.54(0.34–0.86)	0.60(0.28–1.26)
Other	0.74(0.58–0.95)	0.65(0.43–0.99)	0.80(0.61–1.04)	0.79(0.52–1.20)	0.55(0.30–1.01)	0.45(0.16–1.28)
Residence						
Private residence	Reference [1]	Reference [1]	Reference [1]	Reference [1]	Reference [1]	Reference [1]
Nursing home	1.45(0.74–2.83)	2.01(0.58–7.04)	1.14(0.55–2.37)	2.14(0.61–7.54)	4.65(2.03–10.62)	2.26(0.42–12.21)
Homeless	0.80(0.34–1.88)	0.87(0.11–6.93)	0.85(0.34–2.10)	1.48(0.21–10.60)	1.54(0.36–6.51)	1.46(0.09–23.04)
Other	1.42(1.03–1.96)	0.62(0.32–1.22)	1.44(1.03–2.02)	0.67(0.33–1.36)	1.60(0.89–2.90)	0.35(0.08–1.56)
Insurance						
Private insurance	Reference [1]	Reference [1]	Reference [1]	Reference [1]	Reference [1]	Reference [1]
Medicare	0.89(0.67–1.17)	1.16(0.68–1.98)	0.86(0.64–1.16)	0.99(0.56–1.74)	1.14(0.71–1.84)	2.61(1.17–5.81)
Medicaid or CHIP	0.66(0.62–0.70)	0.82(0.73–0.92)	0.74(0.69–0.78)	0.88(0.78–0.99)	0.50(0.44–0.56)	0.75(0.60–0.95)
Uninsured	0.73(0.64–0.82)	0.82(0.65–1.03)	0.76(0.67–0.86)	0.86(0.68–1.09)	0.72(0.56–0.91)	0.80(0.51–1.26)
Other	0.89(0.75–1.05)	1.10(0.79–1.52)	1.00(0.84–1.18)	1.27(0.91–1.76)	0.92(0.68–1.26)	0.70(0.34–1.44)
Arrival by Ambulance						
Yes vs No	1.54(1.39–1.70)	1.19(0.96–1.49)	1.32(1.18–1.46)	1.04(0.83–1.30)	3.70(3.19–4.29)	2.40(1.75–3.31)
Visit year						
2012	Reference [1]	Reference [1]	Reference [1]	Reference [1]	Reference [1]	Reference [1]
2013	0.85(0.79–0.92)	0.73(0.63–0.86)	0.90(0.83–0.97)	0.75(0.63–0.88)	0.70(0.59–0.82)	0.72(0.53–1.00)
2014	0.87(0.81–0.94)	0.79(0.68–0.93)	0.91(0.84–0.99)	0.80(0.68–0.94)	0.77(0.66–0.90)	0.76(0.55–1.04)
2015	0.98(0.90–1.06)	0.89(0.76–1.06)	0.98(0.90–1.07)	0.89(0.75–1.05)	0.89(0.76–1.04)	0.98(0.71–1.34)
2016	0.97(0.90–1.05)	1.01(0.85–1.19)	1.02(0.94–1.11)	1.14(0.96–1.34)	0.75(0.63–0.89)	0.67(0.47–0.94)
Visit month						
Dec-Feb	Reference [1]	Reference [1]	Reference [1]	Reference [1]	Reference [1]	Reference [1]
Mar-May	1.05(0.98–1.13)	1.15(0.99–1.33)	1.03(0.95–1.11)	1.05(0.90–1.22)	1.13(0.97–1.32)	1.46(1.07–1.98)
Jun-Aug	0.94(0.87–1.01)	0.83(0.71–0.97)	0.89(0.82–0.97)	0.77(0.66–0.90)	1.02(0.87–1.20)	0.95(0.68–1.34)
Sep-Nov	1.08(1.01–1.16)	1.00(0.87–1.16)	1.03(0.95–1.11)	0.86(0.74–1.00)	1.21(1.04–1.42)	1.43(1.05–1.93)
Day of Week						
Sunday	Reference [1]	Reference [1]	Reference [1]	Reference [1]	Reference [1]	Reference [1]
Monday	1.05(0.96–1.15)	0.96(0.79–1.15)	1.00(0.91–1.10)	0.91(0.75–1.10)	1.25(1.02–1.54)	0.93(0.63–1.37)
Tuesday	1.06(0.97–1.17)	1.17(0.97–1.42)	1.02(0.93–1.13)	1.20(0.99–1.46)	1.24(1.00–1.53)	0.99(0.67–1.47)
Wednesday	1.03(0.93–1.13)	0.99(0.81–1.20)	0.99(0.89–1.09)	0.93(0.76–1.14)	1.25(1.01–1.54)	1.08(0.73–1.60)
Thursday	1.10(1.00–1.21)	1.01(0.83–1.23)	1.04(0.94–1.16)	0.94(0.77–1.15)	1.31(1.06–1.61)	1.28(0.88–1.86)
Friday	1.10(1.00–1.21)	0.93(0.76–1.14)	1.07(0.96–1.18)	0.98(0.80–1.20)	1.30(1.05–1.61)	0.85(0.56–1.29)
Saturday	1.01(0.92–1.11)	0.84(0.69–1.02)	0.98(0.88–1.08)	0.85(0.70–1.05)	1.21(0.98–1.49)	0.96(0.64–1.44)
Arrival time						
Morning	Reference [1]	Reference [1]	Reference [1]	Reference [1]	Reference [1]	Reference [1]
Afternoon	1.06(0.99–1.15)	0.96(0.82–1.12)	1.06(0.98–1.15)	0.90(0.77–1.06)	1.06(0.91–1.25)	1.18(0.86–1.62)
Evening	1.09(1.00–1.18)	0.87(0.74–1.03)	1.11(1.02–1.21)	0.87(0.74–1.03)	1.01(0.85–1.20)	0.98(0.70–1.37)
Night	0.94(0.87–1.01)	0.89(0.77–1.04)	0.96(0.88–1.04)	0.88(0.75–1.03)	0.96(0.82–1.13)	1.06(0.77–1.46)
Triage level						
Immediate and Emergent	Reference [1]	Reference [1]	Reference [1]	Reference [1]	Reference [1]	Reference [1]
Urgent and Semi-urgent	1.09(0.97–1.23)	1.07(0.86–1.33)	1.07(0.94–1.21)	1.11(0.88–1.40)	0.82(0.67–0.99)	0.93(0.66–1.32)
Nonurgent	0.59(0.53–0.66)	0.53(0.43–0.60)	0.78(0.69–0.88)	0.78(0.62–0.98)	0.21(0.17–0.26)	0.23(0.16–0.33)
Temperature						
36 C-38 C	Reference [1]	Reference [1]	Reference [1]	Reference [1]	Reference [1]	Reference [1]
< =36 C	1.00(0.84–1.17)	0.77(0.55–1.09)	0.93(0.78–1.11)	0.77(0.53–1.10)	1.08(0.78–1.50)	1.21(0.66–2.21)
> 38 C	0.97(0.89–1.06)	1.79(1.48–2.16)	1.18(1.08–1.29)	2.04(1.69–2.48)	0.29(0.21–0.39)	0.67(0.39–1.16)
Diastolic BP						
60–80	Reference [1]	Reference [1]	Reference [1]	Reference [1]	Reference [1]	Reference [1]
< 60	0.60(0.57–0.64)	1.08(0.91–1.28)	0.69(0.65–0.73)	1.01(0.85–1.21)	0.44(0.39–0.50)	1.12(0.81–1.55)
> 80	1.27(1.16–1.38)	1.12(0.94–1.34)	1.17(1.07–1.29)	1.09(0.91–1.31)	1.46(1.25–1.70)	0.99(0.72–1.35)
Systolic BP						
80–120	Reference [1]	Reference [1]	Reference [1]	Reference [1]	Reference [1]	Reference [1]
< 80	0.61(0.58–0.65)	0.83(0.68–1.00)	0.72(0.68–0.77)	0.93(0.76–1.14)	0.35(0.30–0.42)	0.70(0.45–1.08)
> 120	1.49(1.40–1.59)	1.02(0.89–1.18)	1.35(1.26–1.45)	0.99(0.86–1.15)	1.77(1.57–2.00)	1.08(0.83–1.40)
Heart Rate						
60–90	Reference [1]	Reference [1]	Reference [1]	Reference [1]	Reference [1]	Reference [1]
< 60	0.56(0.50–0.61)	1.03(0.78–1.36)	0.64(0.58–0.71)	1.27(0.96–1.68)	0.59(0.49–0.72)	0.90(0.54–1.49)
> 90	0.58(0.55–0.61)	0.89(0.79–1.01)	0.69(0.65–0.74)	1.02(0.89–1.16)	0.43(0.39–0.48)	0.89(0.70–1.13)
Pulse Oximetry						
> =95 vs < 95	0.57(0.50–0.66)	0.61(0.45–0.82)	0.51(0.44–0.59)	0.51(0.38–0.68)	1.05(0.76–1.45)	1.11(0.57–2.18)
Pain level						
Mild	Reference [1]	Reference [1]	Reference [1]	Reference [1]	Reference [1]	Reference [1]
Moderate	2.85(2.64–3.08)	2.15(1.90–2.44)	2.46(2.27–2.67)	1.97(1.73–2.24)	2.48(2.12–2.90)	1.72(1.32–2.22)
Very severe	3.26(2.99–3.56)	2.70(2.32–3.13)	2.72(2.49–2.98)	2.33(2.00–2.72)	3.02(2.56–3.57)	1.94(1.45–2.59)
Injury/poisoning						
Injury/trauma	Reference [1]	Reference [1]	Reference [1]	Reference [1]	Reference [1]	Reference [1]
Overdose/poisoning	0.19(0.14–0.27)	0.18(0.09–0.34)	0.23(0.16–0.33)	0.24(0.12–0.46)	0.21(0.09–0.52)	0.23(0.05–0.96)
Adverse effect of medical/surgical treatment	0.31(0.23–0.41)	0.20(0.11–0.37)	0.29(0.21–0.39)	0.21(0.11–0.41)	0.72(0.44–1.18)	0.67(0.23–1.91)
Not related to any above	0.37(0.35–0.39)	0.32(0.28–0.36)	0.34(0.32–0.36)	0.28(0.24–0.31)	0.43(0.38–0.48)	0.60(0.48–0.76)
Questionable injury status	0.27(0.16–0.48)	0.39(0.10–1.49)	0.22(0.11–0.42)	0.11(0.02–0.90)	0.77(0.31–1.92)	5.68(1.14–28.22)
72 h Revisit						
No vs Yes	1.16(1.01–1.33)	1.11(0.83–1.49)	1.21(1.04–1.41)	1.21(0.89–1.65)	0.97(0.73–1.28)	0.89(0.51–1.56)
Cancer	1.48(0.93–2.36)	1.31(0.30–5.78)	1.35(0.83–2.22)	0.67(0.14–3.24)	2.34(1.12–4.89)	2.85(0.46–17.53)
Cerebral Cardiovascular disease	3.81(1.39–10.48)	–	2.99(1.12–7.98)	–	11.59(4.21–31.93)	–
COPD	2.64(1.88–3.70)	5.85(2.89–11.85)	3.25(2.32–4.56)	7.87(3.91–15.84)	0.14(0.02–1.02)	–
Congestive Heart Failure	3.81(0.91–15.93)	–	2.99(0.75–11.96)	–	2.76(0.34–22.38)	–
HIV	0.71(0.23–2.17)	–	0.64(0.19–2.24)	6.55(0.51–83.75)	1.20(0.16–9.07)	2.81(0.16–50.30)

**Fig. 1:**
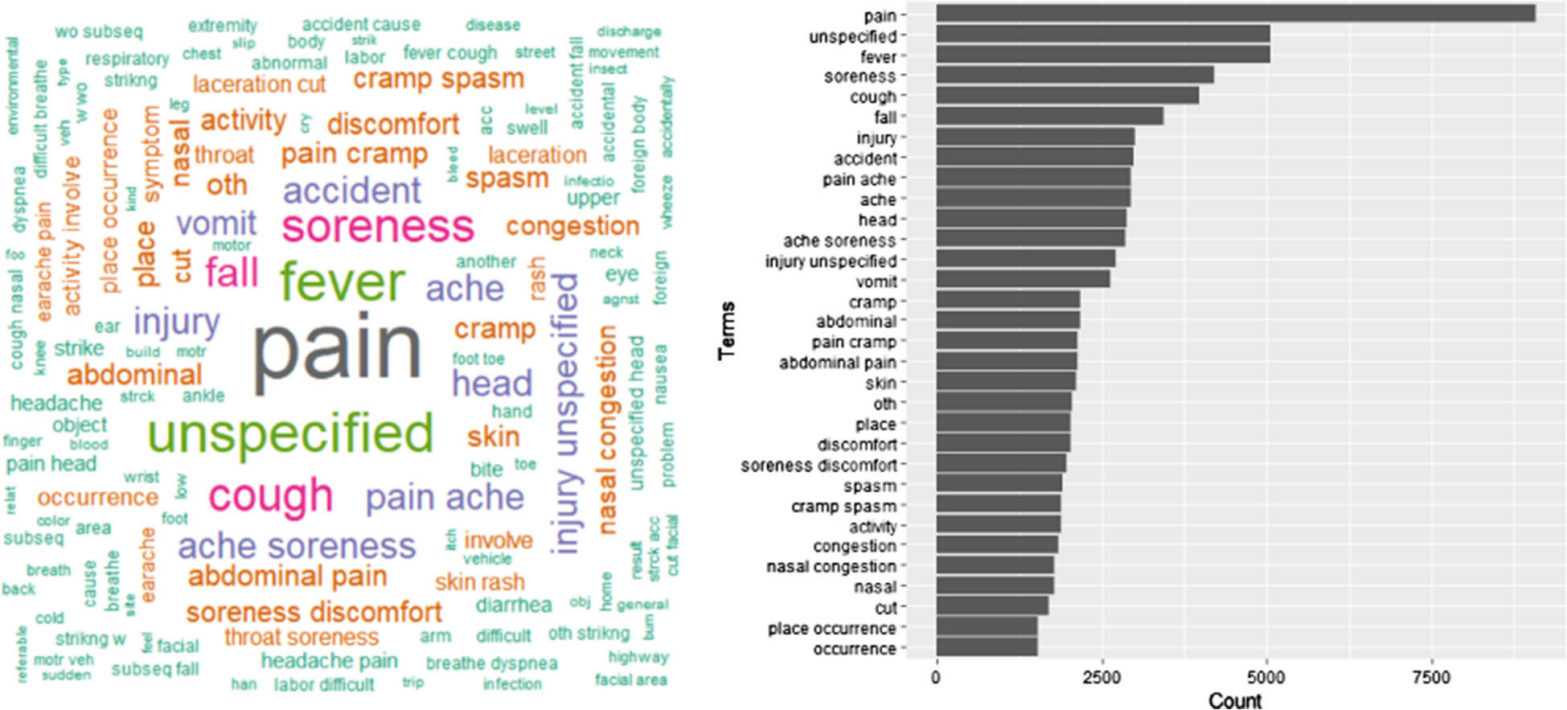
Frequency and the word cloud of the word and word pairs in the unstructured variables (the first figure shows the words that appear over 1500 times, the second shows the words that appear over 20 times)

**Fig. 2:**
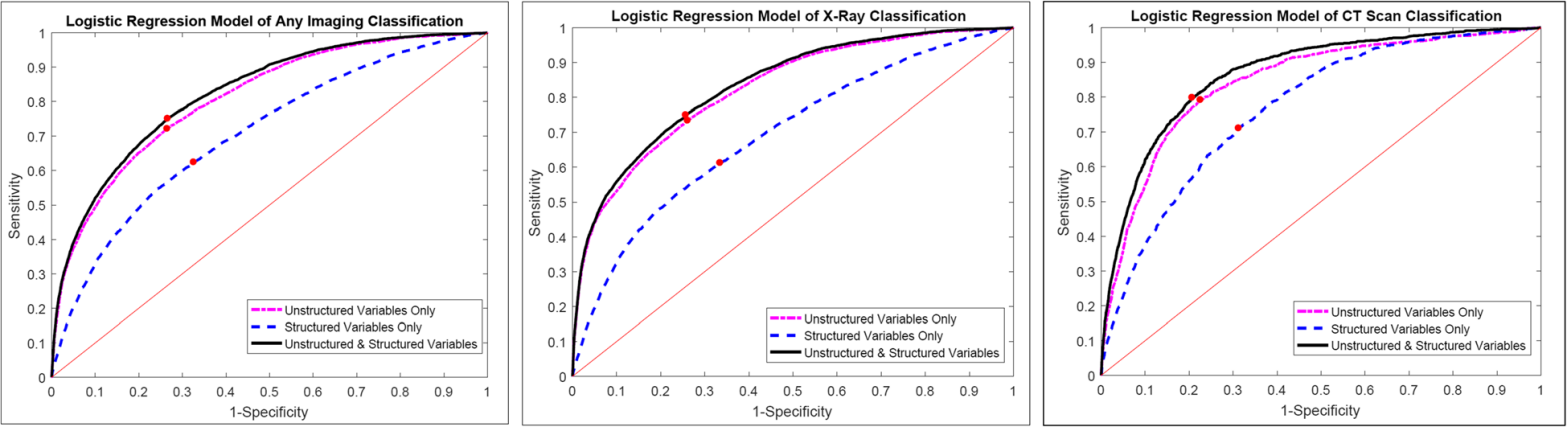
ROC curves for the logistic regression models for medical imaging use (The red point on each ROC curve minimizes the Euclidean distance between the ROC curve and the upper left corner of the coordinate, which is defined as the best cutoff in the study)

The distribution and the odds ratio of the top 25 most frequent words or word pairs were also reported in Fig. 1 and Additional file 1: Table S1. The odds of having imaging were higher for patients whose complaints contained words, such as pain, soreness, injury, and spasm, compared to patients without the presence of those words. Patients reporting fever, vomit, or skin issues showed lower odds of having imaging done. Around 200 principal components remain after feature selection for the input of each logistic regression model. Applying the three logistic regression models (Table 3; model 1: structured variables only, model 2: unstructured variables only, and model 3: both unstructured and structured variables), we found that the predictive accuracy for any medical imaging use was higher for models with text-based reason for visit variables only, compared to models with structured variables only. The AUC (Fig. 2) was 0.71 (95% CI: 0.70–0.71) for any imaging use, 0.69 (95% CI: 0.68–0.70) for X-ray, and 0.77 (95% CI: 0.76–0.78) for CT scan, in the predictive model including only structured variables. Models including only unstructured information obtained *c*-statistics of 0.81 (95% CI: 0.81–0.82) for any imaging use, 0.82 (95% CI: 0.82–0.83) for X-ray, and 0.85 (95% CI: 0.83–0.86) for CT scan. When both structured variables and free text variables were included, the *c*-statistics reached 0.82 (95% CI: 0.82–0.83) for any imaging use, 0.83 (95% CI: 0.83–0.84) for X-ray, and 0.87 (95% CI: 0.86–0.88) for CT scan. The AUC are significantly different between the models on the unstructured data, structured data, and combined data (*p* < 0.001).

**Table 3:** Predictive performance of logistic regression models with 10-fold classification in identifying patients with various medical imaging use during emergency department triage, NHAMCS 2012–2016

	Probability cut-off	Sensitivity	Specificity	Accuracy	AUC (95% CI)
Any Imaging use					
Unstructured variables	0.28	0.72	0.74	0.73	0.810 (0.807–0.813)
Structured variables	0.31	0.62	0.67	0.66	0.706 (0.698–0.714)
Unstructured + Structured variables	0.27	0.75	0.73	0.74	0.824 (0.818–0.829)
Xray					
Unstructured variables	0.22	0.73	0.74	0.74	0.824 (0.822–0.826)
Structured variables	0.26	0.61	0.67	0.65	0.694(0.685–0.704)
Unstructured + Structured variables	0.22	0.75	0.74	0.74	0.834 (0.830–0.839)
CT Scan					
Unstructured variables	0.04	0.79	0.77	0.78	0.845 (0.832–0.858)
Structured variables	0.05	0.71	0.69	0.69	0.771 (0.759–0.783)
Unstructured + Structured variables	0.04	0.80	0.79	0.79	0.868(0.858–0.878)

Note: The best cutoff of the probabilities was determined by using the point on the ROC curve with the shortest distance to the upper left corner (where sensitivity = 1 and specificity = 1)

The result for the sensitivity analysis was reported in Additional file 1: Table S2 and Additional file 1: Figure S1. A number of 420 (1.52% of total) patients had abdomen/pelvis CT scan and 785 (2.84%) had a head CT scan. In the model of abdomen/pelvis CT scan, the AUC was 0.856 (95% CI: 0.833–0.879) for unstructured data, 0.826 (95% CI: 0.814–0.838) for structured data, and 0.892 (95% CI: 0.875–0.909) for both. In the model for head CT scan, the AUC was 0.891(95% CI: 0.877–0.905) for unstructured data, 0.797 (95% CI: 0.786–0.808) for structured data, and 0.906 (95% CI: 0.893–0.920) for both. The AUC are significantly different between the models on the unstructured data, structured data, and combined data (*p* < 0.01).

## Discussion

In the current study, we described the rates of X-Ray use and CT use in pediatric visits to the emergency department in the United States. The rate of medical imaging use ranged from 28.4% to 31.8 each year across from 2012 to 2016; the rate of X-Ray use ranged from 23.8 to 26.2%, and CT’s rate was 4.2 to 5.9%. We found that patients’ socioeconomic, demographic and clinical factors presented at ED triage were associated with the medical imaging use.

Similar to previous studies, we detected racial/ethnic and socioeconomic differences in the use of medical imaging [25, 26]. We found that Blacks and Hispanics were less likely to undergo CT scans compared to white patients, which could be related to the distribution difference of injury severity, or access to insurance coverage, across racial/ethnic groups [25, 27]. Compared to patients with private insurance, patients with Medicaid cover had less likelihood of receiving a CT scan. Reasons for these disparities should be further explored in future research to determine the appropriateness of including or excluding these variables in prediction models [27] based on the clinical context. We also found that younger age, higher triage level, ambulance arrival, abnormal vital signs, injury diagnosis and certain comorbidities were predictive of medical imaging use. As expected, patients with urgent and immediate triage levels had the highest likelihood of medical imaging use. Patients with abnormal vital signs generally had higher likelihood of medical imaging use than the patients with normal vitals.

Clinical practice in adult ED and pediatric ED is largely different, in particular, triaging pediatric patients is more complicated and time-consuming than adults because of their unique physiologic and developmental differences. Compared to our previous study on adult patients, we found even worse racial /ethnic disparities among the black patients compared to white patients in pediatric ED than adult ED. The CT use are positively associated with patients with Medicare in the pediatric patients but opposite for the adult ED patients The CT use are positively associated with urgency of ED among pediatrics.

Since the prediction models are based on the imaging utilization assigned by the clinicians, the associated factors cannot only predict the imaging utilization outcomes but can also indicate the bias in the medical decision in imaging assignment by the clinicians. These biases should be considered in a real implementation of the prediction models in healthcare management. One of the approaches to evaluate these biases would be running a medical chart review from the electronic health records for each patient to analyze how much bias exists in the medical decisions in pediatric ED imaging assignment. Because EDs are the critical staging area for very ill patients, the higher ED utilization and ED overcrowding leads to reduced access to time-critical healthcare, thus negatively affecting patient care quality and patient safety [28–30]. As the crisis of emergency care grows, hospitals have taken initiatives to improve the patient care quality in many ways [31, 32]. One of these is to establish better decision-making systems in emergency care systems that could mitigate these challenges and facilitate the transition to a value-based healthcare industry [33]. Based on large data collected from ED electronic health records and technological innovations that employ predictive analytics to more rapidly identify resources utilization, such as medical imaging.

Prediction models for the adult ED advanced medical imaging utilization (CT, MRI, and ultrasound) has been examined and proposed in a previous study [34]. The main difference between the prediction models in the adult paper and the current study is that single word frequency was used in the adult study for topic modelling, whereas we only kept the first few topics in the prediction models. Topic modelling is a commonly used technique for NLP. Although the method was reported to identify patterns hidden in the unstructured data into different themes, we did not find many clinically meaningful topics when we applied this to the reasons-for-visit data from adult patients. In the current paper using bag-of-words including both single and word pairs, we used a principal component analysis combined with a t-test for the feature extraction. We found that the AUC for pediatric patients (Any imaging use: 0.824; CT scan: 0.868) is improved compared to adults (Any imaging use: 0.780; CT scan: 0.790). The main contributors of the improvement are the bigrams and the inclusion of all features from all bags of words, instead of only keeping the first few. A novel part of this study was the development of a predictive model for medical imaging use among a cohort of pediatric ED patients using both structured and unstructured data available at ED triage. The predictive model showed “good” prediction performance for both medical imaging overall, X-Ray, and CT scan [35]. Although statistically significant, we found that the structured data did not add much prediction power based on the unstructured data in predicting medical imaging utilization for both adult and pediatric patients, indicating that the main factors for imaging utilization at ED were included in the reasons for visit and cause of injury data. A prediction tool built based on the information obtained from patient visits, including the unstructured information written by the triage nurse, may benefit triage personnel and ED physicians, suggesting that the Emergency Severity Index [36–38], a common triage standard in the US, may be underusing the wealth of information available in a typical triage note. Unstructured data from the hospital EHR system have remained largely unexplored as extraction and analysis of these data are complicated [37]. However, information hidden in those unstructured health records provide potentially important information to better predict resource utilization at ED. The prediction improved significantly for all three outcomes when natural-language processing elements were added. The present study adds to similar previous studies [39, 40] by including natural language processing in the ED triage prediction model. Earlier prediction of resource use through tools like those developed here may improve throughput, and improved ED throughput may help reduce ED crowding [32, 34, 41, 42].

The models generally use variables measured at one time point to estimate the probability of an outcome occurring within a given time in the future [43]. Research in prediction models for the ED health service at this stage aims to assist the clinical decision (i.e., to help identify patients’ imaging needs early in the triage period) instead of completely replacing the role of clinicians. Prediction models with good accuracy can efficiently assist the clinical management workflow if there is a good implementation strategy. Medical and economic risk of deploying these models in a real clinical settings is, at this stage, high given the inaccuracies. However, it is still of value to study how to improve the prediction performance, how to better implement those types of prediction tools, and test the values of those models in real implementation, in order to advance the field. This study brought up a new approach to improve the prediction models, and set a base model for imaging prediction at pediatric ED using a national sample. We examined the associated factors of imaging utilization at ED and developed prediction models with good prediction results (AUCs greater than 0.80). Further studies should be performed on how to improve the models’ accuracy, and how to implement the models with good accuracy as well as assess the medical-economic risk.

### Limitations and strengths

This study is limited in several ways. Limits of the data source (NHAMCS) include that (1) the outcomes of medical imaging use are based on clinical decisions made with awareness of the predictors used in the model, with resulting incorporation bias [44]; (2) the survey did not collect the information of the subtype of X-rays, or (3) information on the appropriateness of imaging utilization and pediatric specific comorbidities; (4) the survey rely on clinician diagnoses and it is not possible to validate the diagnoses; (5) NHAMCS uses visits and not individual patient counts, so it is possible that some children had multiple visits, or received multiple imaging, particularly those more medically complex. The NLP approach simplified the feature extraction using the frequency of word and word pairs existing in the text data. The approach ignored other information, such as word combination with more than bigrams, or the order of the words, which could exclude specific predictors. However, the number of words was small within each text field, so we would expect to capture clinically relevant information by simply extracting the frequency of the words and word pairs.. Limitations of the use of the c-statistic: it is a single number and summarizes the discrimination of a model but does not communicate all the information ROC plots contain and lacks direct clinical application [43].

Strengths of the NHAMCS include national representativeness, increasing the generalizability of the data. This study was based on retrospective national survey samples, and should be viewed as preliminary in the hierarchy of diagnostic test validity. Future perspective studies should be performed to test the effectiveness of the predictive models.

## Conclusions

Using a nationally representative data of pediatric patients presenting to the ED, we examined information relating to the patients’ socioeconomic, demographic and clinical factors during the patients’ ED visits, including unstructured free-text fields such as the reason for visiting, and developed predictive models for medical imaging use. Both CT and X-rays are commonly used in the pediatric ED with one third of the visits receiving at least one. We present several predictive models for the use of medical imaging in pediatric patients visiting the ED. The inclusion of unstructured data (ie: triage notes) provided significant improvement in accuracy.

## Supplementary information

**Additional file 1: Table S1.** Characteristics of the top 25 most frequent words in the patient complaint and cause of injury by imaging use. **Table S2.** Predictive performance of logistic regression models with 10-fold classification in identifying patients with abdomen/pelvis and head CT scan during emergency department triage, NHAMCS 2012–2016. **Figure S1.** ROC curves for the logistic regression models for abdomen/pelvis and head CT scan (The red point on each ROC curve minimizes the Euclidean distance between the ROC curve and the upper left corner of the coordinate, which is defined as the best cutoff in the study).

## Data Availability

The NHAMCS-ED dataset can be accessed through the website of the US Centers for Disease Control and Prevention (CDC) (https://www.cdc.gov/nchs/ahcd/index.htm). The detailed explanation of the survey data for each year and the code book can be found here: https://ftp.cdc.gov/pub/Health_Statistics/NCHS/dataset_documentation/nhamcs/ The SAS dataset for each year can be found here: https://ftp.cdc.gov/pub/Health_Statistics/NCHS/Datasets/NHAMCS/ All the data were in a SAS format. To get the unstructured data, one needs to run the SAS format files under the following link before import the data to the analysis software. https://ftp.cdc.gov/pub/Health_Statistics/NCHS/dataset_documentation/nhamcs/sas/

## Abbreviations

aOR: Adjusted Odds Ratio
AUC: Area under Curve
CI: Confidential Interval
CT: Computed tomography
ED: Emergency Department
EHR: Electronic Health Record
MRI: Magnetic Resonance Imaging
NHAMCS: National Hospital Ambulatory Medical Care Survey ED Subfile
NLP: Natural Language Processing
PCA: Principal Component Analysis
ROC: Receiver Operating Curve
