# Supplementary material for: Use of natural language processing to improve predictive models for imaging utilization in children presenting to the emergency department
Source: BMC Med Inform Decis Mak. 2019 Dec 30;19:287. doi: 10.1186/s12911-019-1006-6 (PMC6937987; doi:10.1186/s12911-019-1006-6)

Supplement Table 1 Characteristics of the top 25 most frequent words in the patient complaint and cause of injury by imaging use.

|  | All | No imaging | Any Imaging | Crude Odds Ratio |
| --- | --- | --- | --- | --- |
| pain | 7,945(28.7) | 4,346(22.6) | 3,599(42.9) | 2.58(2.44-2.72) |
| fever | 5,045(18.2) | 3,825(19.8) | 1,220(14.5) | 0.69(0.64-0.74) |
| unspecified | 4,503(16.3) | 2,305(12.0) | 2,198(26.2) | 2.61(2.45-2.79) |
| soreness | 3,961(14.3) | 1,934(10.0) | 2,027(24.1) | 2.85(2.66-3.06) |
| cough | 3,977(14.4) | 2,800(14.5) | 1,177(14.0) | 0.96(0.89-1.03) |
| fall | 3,317(12.0) | 1,585(8.2) | 1,732(20.6) | 2.90(2.70-3.12) |
| injury | 2,850(10.3) | 1,140(5.9) | 1,710(20.4) | 4.07(3.76-4.41) |
| accident | 2,745(9.9) | 1,532(7.9) | 1,213(14.5) | 1.96(1.81-2.12) |
| ache | 2,699(9.8) | 786(4.1) | 1,913(22.8) | 6.94(6.36-7.58) |
| pain ache | 2,699(9.8) | 786(4.1) | 1,913(22.8) | 6.94(6.36-7.58) |
| head | 2,718(9.8) | 1,887(9.8) | 831(9.9) | 1.01(0.93-1.10) |
| ache soreness | 2,627(9.5) | 757(3.9) | 1,870(22.3) | 7.01(6.41-7.66) |
| injury unspecified | 2,600(9.4) | 961(5.0) | 1,639(19.5) | 4.62(4.25-5.03) |
| vomit | 2,630(9.5) | 1,923(10.0) | 707(8.4) | 0.83(0.76-0.91) |
| cramp | 2,140(7.7) | 1,176(6.1) | 964(11.5) | 2.00(1.83-2.18) |
| abdominal | 2,136(7.7) | 1,176(6.1) | 960(11.4) | 1.99(1.82-2.17) |
| pain cramp | 2,108(7.6) | 1,153(6.0) | 955(11.4) | 2.02(1.84-2.21) |
| abdominal pain | 2,107(7.6) | 1,153(6.0) | 954(11.4) | 2.02(1.84-2.21) |
| skin | 1,845(6.7) | 1,742(9.0) | 103(1.2) | 0.13(0.10-0.15) |
| oth | 1,983(7.2) | 1,214(6.3) | 769(9.2) | 1.50(1.37-1.65) |
| place | 1,823(6.6) | 887(4.6) | 936(11.2) | 2.60(2.36-2.86) |
| discomfort | 1,896(6.9) | 546(2.8) | 1,350(16.1) | 6.57(5.93-7.29) |
| soreness discomfort | 1,841(6.7) | 521(2.7) | 1,320(15.7) | 6.72(6.05-7.46) |
| spasm | 1,872(6.8) | 988(5.1) | 884(10.5) | 2.18(1.98-2.40) |
| cramp spasm | 1,864(6.7) | 983(5.1) | 881(10.5) | 2.18(1.98-2.40) |

Supplement Table 2. Predictive performance of logistic regression models with 10-fold classification in identifying patients with abdomen/pelvis and head CT scan during emergency department triage, NHAMCS 2012-2016

|  | Probability cut-off | Sensitivity | Specificity | Accuracy | AUC (95% CI) |
| --- | --- | --- | --- | --- | --- |
| **CT Scan-Abdomen/pelvis** |  |  |  |  |  |
| Unstructured variables | 0.01 | 0.78 | 0.86 | 0.86 | 0.856 (0.833-0.879) |
| Structured variables | 0.02 | 0.77 | 0.75 | 0.75 | 0.826 (0.814-0.838) |
| Unstructured + Structured variables | 0.01 | 0.83 | 0.85 | 0.85 | 0.892 (0.875-0.909) |
| **CT Scan-Head** |  |  |  |  |  |
| Unstructured variables | 0.02 | 0.83 | 0.84 | 0.84 | 0.891(0.877-0.905) |
| Structured variables | 0.03 | 0.74 | 0.70 | 0.70 | 0.797 (0.786-0.808) |
| Unstructured + Structured variables | 0.02 | 0.85 | 0.84 | 0.84 | 0.906 (0.893-0.920) |

Note: The best cutoff of the probabilities was determined by using the point on the ROC curve with the shortest distance to the upper left corner (where sensitivity=1 and specificity=1).

Supplement Figure 1. ROC curves for the logistic regression models for abdomen/pelvis and head CT scan (The red point on each ROC curve minimizes the Euclidean distance between the ROC curve and the upper left corner of the coordinate, which is defined as the best cutoff in the study)


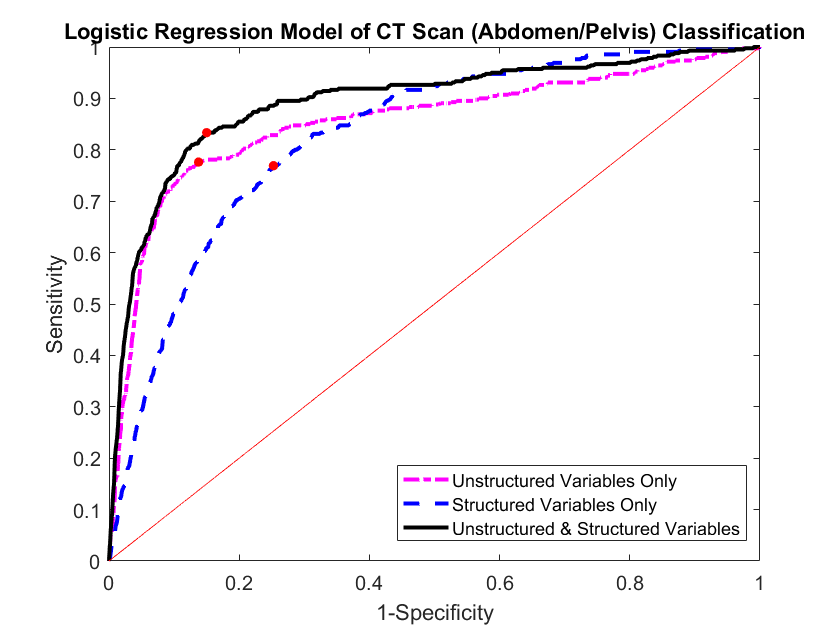

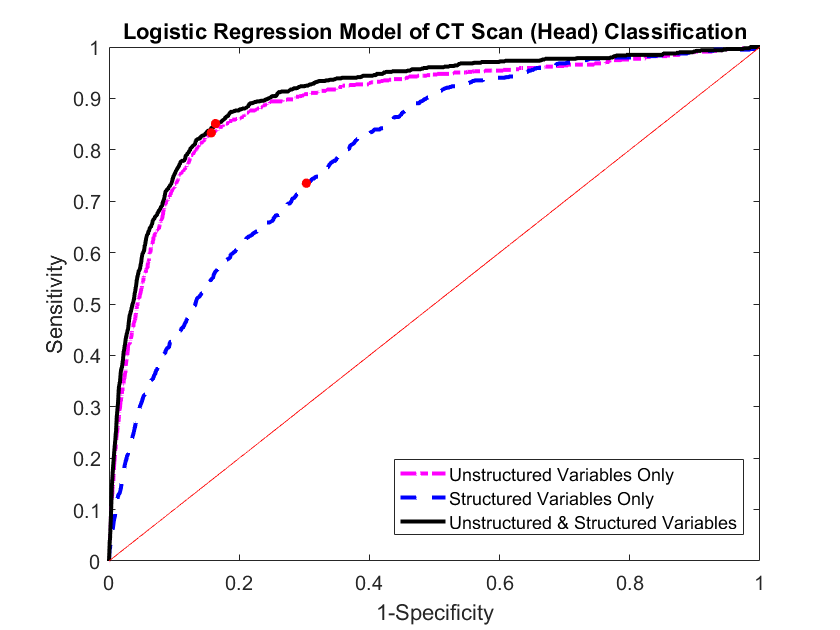

Supplement: Supplementary file 1 — Additional file 1: Table S1. Characteristics of the top 25 most frequent words in the patient complaint and cause of injury by imaging use. Table S2. Predictive performance of logistic regression models with 10-fold classification in identifying patients with abdomen/pelvis and head CT scan during emergency department triage, NHAMCS 2012–2016. Figure S1. ROC curves for the logistic regression models for abdomen/pelvis and head CT scan (The red point on each ROC curve minimizes the Euclidean distance between the ROC curve and the upper left corner of the coordinate, which is defined as the best cutoff in the study). [file 12911_2019_1006_MOESM1_ESM.docx]
